# The data-hypothesis conversation

**DOI:** 10.1186/s13059-021-02277-3

**Published:** 2021-02-10

**Authors:** Itai Yanai, Martin Lercher

**Affiliations:** 1 Institute for Computational Medicine, NYU Langone Health, New York, NY 10016 USA; 2 Institute for Computer Science & Department of Biology, Heinrich Heine University, 40225 Düsseldorf, Germany

The greatest value of a picture is when it forces us to notice what we never expected to see.

John W. Tukey, Exploratory Data Analysis, 1977

Where do ideas come from? Modern science has revealed humanity to be located in a mediocre part of the universe (not at its center), as one of millions of evolved species (not uniquely created), and with a genome smaller than that of an onion. Yet, perhaps in an attempt to hold on to a specter of a special position in the universe, some thinkers cling on to the notion that humanity is endowed with the wisdom to intuit new insights by philosophizing, independent of data and observations. But scientific ideas and hypotheses do not simply descend upon the human mind, fully formed and ready for day science testing. In our Genome Biology series, we attempt to demystify the processes of creative and exploratory thinking that lead to scientific ideas and hypotheses in what may be called “night science” [1]. Recently, we argued that when we approach a dataset with the intent of testing a specific hypothesis, the corresponding mindset may get in the way of unexpected discoveries. In this important sense, a hypothesis can be a liability [2]. The alternative mindset associated with exploratory data analysis has its own liabilities, but together, day science testing and complementary night science explorations fuel science’s unique power for discovery.

In response to our arguments, a group of authors has issued a critique with the central objection that our notions ignore a “temporal primacy of the hypothesis,” meaning that data only makes sense in the light of hypotheses [3]. What these authors—Felin, Ellis, Noble, Koenderink, and Krueger (whom we acronym as FENKK)—overlook, however, is that the entirety of modern science is one long conversation not only between scientists, but, more deeply, between data and hypotheses [4]. One project’s hypothesis comes from the last project’s data and results, be they our own or someone else’s. To paraphrase Leonard Cohen, science is not a victory march of reason— it is a cold and it is a broken conversation with data.

## The unbearable lightness of the “proto-hypothesis”

FENKK posit that it is logically impossible to make observations without theory, arguing against the possibility of hypothesis-free data exploration. To illustrate this position, imagine that you are investigating the cell types and cell states present in the blood during infection by a specific pathogen. You should have a good understanding of what is already known, including the erythrocytes, the monocytes, the macrophages, and their many recognized states. This conceptual and theoretical backdrop of the expected will be crucial for your ability to recognize when something unexpected may be at hand. Moreover, in order to make sense of any data you generate, you should know the ins and outs of the experimental techniques and the data analysis tools that you are using. No one would argue with this notion that the underlying theoretical backdrop of a problem and its associated data are a necessary prerequisite for any scientific exploration. We had in fact highlighted this in our paper, writing that one needs to contrast “any observed pattern on an elaborate mental background that represents the expected” [2]. To successfully explore a dataset, this mental background should be as comprehensive as possible.

Does the necessity of a theoretical background then mean that it is logically impossible to explore datasets in a hypothesis-free mode, as we recommend in our paper? FENKK argue that it does—but do so using a trick. They change the very definition of the term “hypothesis,” making it absurdly broad. Their broadened term—for which they invent the interchangeable label “proto-hypothesis”—encompasses any “latent expectation, question or even guess about what might be lurking, about what might potentially be interesting or relevant, and how it might be caught.” With this redefinition, “hypothesis” becomes a synonym for mental constructs, meaning that no conscious human activity can ever be hypothesis-free. But through “[...] stretching the definition of a hypothesis by including expectations, conjecture and even the statistical and computational tools that are used to generate insight” [3], FENKK completely miss the point of what hypotheses mean to science.

For us and the rest of the scientific community, a hypothesis is the idea that one sets out to test. In contrast, the innumerable theories that underpin our conception of the world in general and the topic we are studying specifically form the background of our analysis or exploration. As one analyzes a dataset with a particular conceptual background, we argue that one can do so productively without a specific hypothesis—indeed, this very notion forms the basis of the field of exploratory data analysis [5]. When a discovery leads to a new idea, this has to be transformed into a formal hypothesis so that it can be tested in the objective light of day science; but the idea had to first emerge. Our argument is simply that if you have an information-rich dataset, it will often be beneficial to analyze it with an open mind instead of with (or in addition to) the express or even pre-registered intention to test a specific hypothesis. In that sense, setting out to test a hypothesis may turn out to be a liability.

## “Hypothesis primacy” as an ahistorical view of science

In response to our promotion of hypothesis-free data exploration, FENKK accuse us of “ignor [ing] the temporal primacy of theory and hypothesis”, illustrating their point with a simple figure that connects the words “Wisdom,” “Knowledge,” “Information,” and “Data” [3]. But anyone who has ever done a scientific experiment knows that the actual hypothesis they set out to test is never pulled from the ether. It is hard won from data. If hypotheses were routinely generated by data-free philosophizing, we would still be doing natural philosophy in the style of Aristotle [4]. Essentially, what FENKK’s hypothesis-first notion misses is this: just as much as data is built on ideas, ideas themselves are built on data. Together, data and hypothesis form an eternal exchange going back and forth, best described as a conversation.

In 1985, Denis Noble (the “N” in “FENKK”) and his colleague Dario DiFrancesco published an influential model of cardiac rhythms [6]. Did the authors arrive at this model starting from theory? As disclosed in Denis Noble’s book “Dance to the tune of life” [7] and in a recent review [8], the actual process was more interesting—and, we argue, representative of the day science/night science conversation of data and hypotheses across individual projects. A few years earlier, Susan Noble (Denis Noble’s wife), Dario DiFrancesco, and Hilary Brown had observed a particular current in the heart with an unexpected mode of activation [9], naming this new current *i*_f_. As Denis Noble explains, this naming followed “... the lab jargon, which dubbed it the ‘funny’ current. Why ‘funny’ [...]? There was a niggling doubt about its origin. It was called ‘funny’ because of that doubt” [7].

That is, the measurements made by Susan Noble and her co-workers revealed something to be explained. Had this “funnyness” of the data been part of a pre-experimental hypothesis? No. Was its revelation a product of theory? No. There was an underlying notion of what “normal” currents in the heart ought to look like, but that was background knowledge; it was not what these scientists had set out to test. The data led to more questions than it answered, the hallmark of any great dataset. Denis Noble continues, talking about the molecular origin of the current: “This little gap in the evidence widened to a huge chasm when, a year later, Dario proved it to be a different class of protein channel altogether.” They realized that, somehow, a sodium current had masqueraded as a potassium one. With this data at hand, Noble and DiFrancesco finally had what they needed to build a model: “For the next five years, he and I worked to completely reformulate the mathematical theory of cardiac rhythm” [7].

**Fig. 1:**
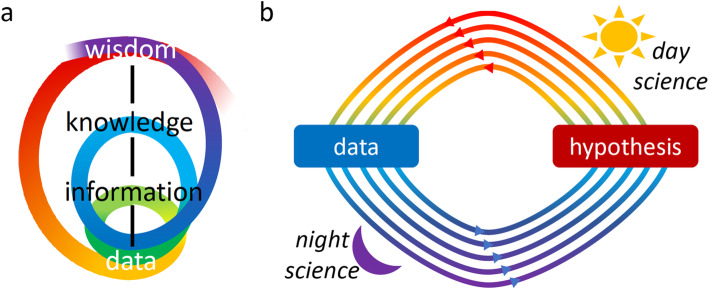
The data-hypothesis conversation. **a** The actual path to discovery (indicated with changing colors) is more convoluted than the simple DIKW model (cited by FENKK [3]) suggests. **b** The data-hypothesis conversation can be abstracted as a spiral occurring over iterative periods of day science and night science [1]

As illustrated by this example, the process that leads to knowledge follows a convoluted path, where one project’s data leads to the next project’s hypothesis (see Fig. 1b). To talk of a hypothesis primacy for any of the three projects that culminated in the discovery of the funny current, the sodium channel, and the model, respectively, is to selectively forget the path that led to each project. While it may be esthetically pleasing to publish a paper that introduces one’s own contribution with “We hypothesized … ”, this version of events sweeps the often interesting prequel to the experiment under the carpet. Far too rarely, the full stories are unveiled when scientists describe the antecedents in their autobiographies.

To give another example, consider a recent project in which one of us worked with a team studying tumorigenesis [10]. Based upon reports of cancer cell states, we began by asking if we could observe tumor evolution in terms of a progression through these states over time, collecting data with that goal in mind. Analyzing the results, however, we did not detect much evidence for changes in state frequencies. So much for our original hypothesis. What the data did show, though, was unexpected evidence for an unusual, not well-characterized state. Continuing along with the data-hypothesis conversation, we decided to focus on that state and generated more data to validate its existence, and we sought out insights into its possible role in the tumor. Figure 1a, which is based upon the linear top-down/bottom-up schematic reproduced by FENKK, shows a map of the convoluted progression of our project: while a question or hypothesis drove each collection of data, each new question or hypothesis was in turn triggered by the analysis of an earlier dataset.

In support of the notion that “many of the greatest insights have come top-down, where scientists start with theories and hypotheses that guide them to identify the right data and evidence”, FENKK refer to Einstein’s theory of special relativity, suggesting that Einstein developed this theory by “quer [ing] fundamental assumptions that are taken for granted, such as [...] that simultaneity is independent of the state of motion.” But that is not at all how it happened. Before his discovery of special relativity, Einstein had spent years unsuccessfully trying to modify Maxwell’s equations so that they would become consistent with unexplained data. Only when that failed, he finally realized that the problem was not with Maxwell, but that the very concept of time itself had to be changed [11]. Thus, rather than constituting a profound example of the primacy of the hypothesis, the theory of special relativity is the fruit of a drawn-out data-hypothesis conversation (Fig. 1b).

## Gorillas and other distractions

We have argued that the two distinct modes of science each have their own liabilities and assets [2]. Night science generates ideas through the exploration of data and conceptual backgrounds, but leads to many false ideas and blind alleys. Day science, on the other hand, can test ideas conclusively, but, we hypothesized, may bias you against making discoveries within the reach of your dataset. To test our hypothesis, we devised an experiment where we gave two groups of students the same data but different assignments: while the “hypothesis-free” group was simply asked to analyze the data and to report their conclusions, the “hypothesis-focused” group was additionally asked to test three specific hypotheses about differences in means and correlations. However, the dataset was one we simply made up so that it revealed a clear and unmistakable image of a gorilla when the two main features—number of steps taken on a particular day and the body mass index—were plotted against each other. As expected from our conjecture, “hypothesis-free” students were almost five times more likely to observe the gorilla than “hypothesis-focused” students.

FENKK criticize our study design as an example of attentional misdirection, where an experiment is purposely designed such that subjects are distracted and therefore miss otherwise obvious visual cues. We designed our experiment as a homage to the classic invisible gorilla experiment, in which viewers miss a gorilla when they are distracted by counting basketball passes [12]. We venture that FENKK were drawn to write their critique because of this choice, as three of the authors had earlier written a critique of the classic study, referring to it as “surprise hacking” [13]. In the same vein, here they are arguing that we set up an artificial situation engineered to distract the students, just as magicians do with sleight of hand tricks.

This is a case where a mere copy-paste of a critique of one experiment does not transfer to another. Independently of how valid their critique of the classic study may have been, FENKK’s error here is to miss that setting up hypotheses as a distraction was not a design flaw of our study. Instead, it was an essential feature: we wanted to test whether having specific hypotheses would indeed act as a distraction. We see such distractions as liabilities for carrying out unbiased conversations with the data. The gorilla was simply a tool for registering whether students plotted the data, which, as we wrote, is the first simple step towards data exploration [2]. The shape of a cloud may be irrelevant to a meteorologist, as argued by FENKK. But for scientists analyzing their data, the shapes of clouds of data points are all but irrelevant: they guide us to the identification of clusters [5], to the effects of outliers [14], and to the dependencies of trends on groupings (as in Simpson’s paradox [15]), all of which require the plotting of the data.

## The eternal conversation

What is perhaps most striking about FENKK’s response is its complete neglect of the very context under which our paper is to be read. In our *Genome Biology* series, our premise has been that one important first step in making discoveries is to make a conscious distinction between day science, where ideas are turned into hypotheses and tested, and night science, where ideas are born [1]. We argue that while a full comprehension of scientific creativity (or any other type of creativity, for that matter) may be out of reach, there are patterns, principles, and tricks that can assist the creative process. In previous articles, we discussed the two languages of day and night science [16], the role of interdisciplinary thinking [17], and how the formulation of new questions often precedes new hypotheses [18].

While we wrote about night science and hypothesis-free explorations, we gladly admit that hypotheses occupy a central space in day science. Any hypothesis, whether divined by wisdom or spawned by a fishing expedition, must be subjected to rigorous attempts at falsification, and this is clearly the domain of day science. What we mean to suggest is that the hypothesis-testing part is only half of the process; the other half, comprising the untold story of how hypotheses are generated, deserves the same attention. Science owes much of its progress to serendipity—to unexpected, unplanned findings. Data exploration beyond specific hypotheses may increase our chances to stumble upon such serendipitous discoveries.

Freed from semantics and reduced to its essence, the debate between us and FENKK boils down to the question of what comes first: the data or the hypothesis, day science or night science. While FENKK argue for a dominant role of hypothesis first, we stress that there cannot be primacy of one or the other. One project’s hypothesis comes from last project’s data and results. Over time, hypotheses and explorations form an eternal conversation, leading from prehistory to the “knowledge machine” that science is today [4].
